# Adipose-Derived Mesenchymal Stem Cells Modulate Fibrosis and Inflammation in the Peritoneal Fibrosis Model Developed in Uremic Rats

**DOI:** 10.1155/2020/3768718

**Published:** 2020-05-20

**Authors:** Elerson C. Costalonga, Camilla Fanelli, Margoth R. Garnica, Irene L. Noronha

**Affiliations:** Laboratory of Cellular, Genetic and Molecular Nephrology, Renal Division, University of São Paulo, São Paulo, Brazil

## Abstract

Peritoneal fibrosis (PF) represents a long-term complication of peritoneal dialysis (PD), affecting the peritoneal membrane (PM) function. Adipose tissue-derived mesenchymal stem cells (ASC) display immunomodulatory effects and may represent a strategy to block PF. The aim of this study was to analyze the effect of ASC in an experimental PF model developed in uremic rats. To mimic the clinical situation of patients on long-term PD, a combo model, characterized by the combination of PF and chronic kidney disease (CKD), was developed in Wistar rats. Rats were fed with a 0.75% adenine-containing diet, for 30 days, to induce CKD with uremia. PF was induced with intraperitoneal injections of chlorhexidine gluconate (CG) from day 15 to 30. 1 × 10^6^ ASC were intravenously injected at days 15 and 21. Rats were divided into 5 groups: control, normal rats; CKD, rats receiving adenine diet; PF, rats receiving CG; CKD+PF, CKD rats with PF; CKD+PF+ASC, uremic rats with PF treated with ASC. PF was assessed by Masson trichrome staining. Inflammation- and fibrosis-associated factors were assessed by immunohistochemistry, multiplex analysis, and qPCR. When compared with the control and CKD groups, GC administration induced a striking increase in PM thickness and inflammation in the PF and CKD+PF groups. The development of PF was blocked by ASC treatment. Further, the upregulation of profibrotic factors (TGF-*β*, fibronectin, and collagen) and the increased myofibroblast expression observed in the CKD+PF group were significantly ameliorated by ASC. Beyond the antifibrotic effect, ASC showed an anti-inflammatory effect avoiding leucocyte infiltration and the overexpression of inflammatory cytokines (IL-1*β*, TNF-*α*, and IL-6) in the PM induced by GC. ASC were effective in preventing the development of PF in the experimental model of CKD+PF, probably due to their immunomodulatory properties. These results suggest that ASC may represent a potential strategy for treating long-term PD-associated fibrosis.

## 1. Introduction

Peritoneal dialysis (PD) is a safe life-sustaining renal replacement modality, employed for the treatment of end-stage renal disease (ESRD) worldwide. According to the last registry, 11% of the global dialysis population is under PD [1–3]. In many countries, patient outcomes with PD are comparable to or better than those with hemodialysis, and PD is also more cost effective.

In spite of providing the best preservation of residual renal function and higher quality of life for patients, compared to hemodialysis, PD promotes continuous exposure of the peritoneal membrane (PM) to bioincompatible, hypertonic dialysis solutions, which can cause chronic PM inflammation. Moreover, PD patients are under the risk of infectious peritonitis. The long-term exposure to PD fluids associated with recurrent episodes of infectious peritonitis induces inflammation, neoangiogenesis, and peritoneal fibrosis (PF), which impairs its function, leading to technical failure of this modality [4, 5].

The pathophysiology of PF involves the loss of mesothelial cells and the thickening of the submesothelial area, mainly composed of an extracellular matrix (ECM) and myofibroblasts. Recent studies have proposed that mesothelial cells represent an important source of myofibroblasts, through the epithelial–mesenchymal transition. However, a recent study showed the important role of submesothelial resident fibroblasts as myofibroblast precursors in PF [6]. Besides myofibroblasts, submesothelial infiltration by leukocytes, such as macrophages and T-cells, is also usually observed. The activation of these inflammatory cells is driven by irritative stimuli, such as the high concentrations of glucose and glucose-degradation products found in the dialysis fluid, which start to synthesize and release a number of proinflammatory factors, namely, the IL-1-*β*, TNF-*α*, IL-6, and specially, the transforming growth factor-*β* (TGF-*β*). Under TGF-*β* signaling, myofibroblasts, characterized by *α*-SMA expression, produce ECM proteins, such as collagen and fibronectin, leading to the development of PF [7, 8].

Current strategies to minimize PF in patients submitted to PD include the use of bioincompatible dialysis solutions. Additionally, clinical administration of antifibrotic drugs, such as tamoxifen, has been described in some patients as an attempt to abrogate peritoneal inflammation and fibrosis. However, these approaches are only partially effective [7, 9, 10]. Furthermore, experimental blockade of inflammation and TGF-*β* by the administration of valproic acid, tamoxifen, and bone morphogenetic protein-7 (BMP7) have shown positive effects in preventing PF progression in animal models. Nevertheless, further studies are required to confirm the efficiency and safety of these compounds [5, 10].

In this context, the research for alternative approaches to prevent PF, such as cell-based therapy, is of paramount importance. Previous experimental studies demonstrated that administration of mesenchymal stem cells (MSC) promotes renoprotection by preventing the development of renal inflammation and fibrosis in models of both acute and chronic kidney disease (CKD), due to its immunomodulatory effects [11, 12]. Since there are expressive similarities between the mechanisms of renal and peritoneal fibrogenesis, the aim of the present study was to analyze the potential anti-inflammatory and antifibrotic effects of adipose-derived MSC (ASC) administration in rats submitted to a combined model of uremic CKD+PF, which better reproduces the pathophysiological scenario of long-term PD.

## 2. Materials and Methods

### 2.1. Animal Model

Thirty-eight adult male Wistar rats weighing 300-350 g were obtained from the local animal facility of the University of São Paulo (USP). Animals were kept at a constant temperature of 23 ± 2°C, under a 12 h light/dark cycle and had free access to tap water. All animal procedures were approved by the Research Ethics Committee of USP Faculty of Medicine (FMUSP-CAPPesq 029/2016) and were conducted in accordance with our institutional guidelines and with international regulations for manipulation and care of experimental animals. In order to mimic the clinical situation of patients on long-term PD, a combo model, characterized by the combination of PF and uremia, was employed in the present study [10]. Uremia was induced by an adenine-rich diet. Twenty-four animals were fed a 0.75% adenine-containing rat diet (Sigma Co., St. Louis, USA) for 30 consecutive days, while the 14 remaining animals were fed with standard rat chow (Nuvital Labs, Curitiba, Brazil). PF was induced in 24 animals by IP injections of chlorhexidine gluconate (CG). Body weight was assessed once a week, and tail-cuff systolic blood pressure was measured in conscious animals with an automated optoelectronic device (Visitech Systems, USA), at the end of the study period.

### 2.2. Experimental Protocol

After 15 days of adenine-rich diet administration, when uremia was already established, PF was induced by daily IP injections of CG. Two intravenous (IV) doses of 1 × 10^6^ ASC each were administered to the treated group at two different moments. The first dose of ASC was given concomitantly with the first IP CG injection (15 days after the adenine-rich diet administration began). The second dose was given 6 days later, 21 days after the adenine-rich diet administration began. All animals were studied for a total of 30 days. Our experimental protocol consisted of the following groups:

- CKD: animals receiving adenine-rich diet for 30 days to induce severe CKD (*N* = 8)
- PF: animals fed with standard rat diet, submitted to the CG-induced PF model (*N* = 8)
- CKD+PF: CKD animals submitted to the CG-induced PF model 15 days after the adenine-rich diet administration began (*N* = 8)
- CKD+PF+ASC: CKD+PF animals which received 2 IV infusions of 1 × 10^6^ ASC each, diluted in sterile PBS. The first infusion was performed concomitantly with the first CG IP injection, 15 days after the adenine-rich diet administration began, and the second one was performed 21 days after the adenine-rich diet administration began (*N* = 8)
- Control: animals fed with standard rat diet and kept untreated for 30 days (*N* = 6).

### 2.3. Isolation, Expansion, and Characterization of Rat ASC

Gonadal adipose tissue from 5 healthy adult male Wistar rats was obtained after its euthanasia with an IP injection of 0.1 g of sodium thiopental. The adipose tissue samples were minced with sterile scissors and digested in a 0.075% collagenase solution (Sigma-Aldrich, USA). After centrifugation, the isolated cells were cultured under 37°C and 5% CO_2_ in plastic culture flasks with Dulbecco's Modified Eagle Medium (DMEM-low glucose, Invitrogen, USA) containing 10% inactivated fetal bovine serum (FBS; Invitrogen), 100 units/mL penicillin, and 100 mg/mL streptomycin antibiotic solution (Gibco, Carlsbad, MO, USA). Culture medium was changed three times a week, and cells were trypsinized and reseeded when they reached 80% of confluence. At the 4^th^ passage, cells were characterized as MSC according to the criteria defined by the International Society of Cellular Therapy Consensus: adherence to plastic under standard conditions, positivity to specific surface markers, such as CD29, CD44, CD90, and CD105; negativity to CD45; and ability to differentiate into mesenchymal lineages when submitted to appropriate culture medium and stimuli.

**Figure 1:**
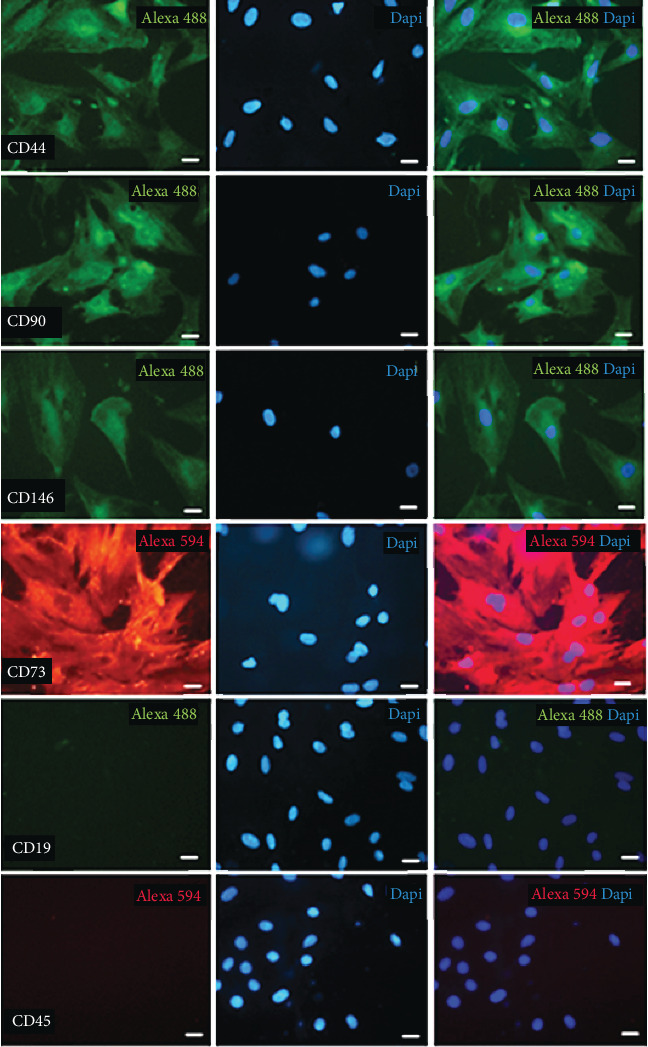
Cellular characterization of mesenchymal stem cell surface markers by immunofluorescence. ASC were positive for CD44, CD90, CD146, and CD73 and negative for CD19 and CD45.

Cells were used at passages 4-6 according to flow cytometry analyses (FACSCanto™, BD Biosciences, USA). For this purpose, ASC were labeled with isothiocyanate- (FITC-) conjugated antibodies against CD31, CD29, and CD90; phycoerythrin- (PE-) conjugated antibodies against CD34, CD44, and CD105; Pe-cy5.5-conjugated antibody against CD45; and FITC- or PE-conjugated nonspecific IgG (eBioscience, San Diego, USA). The results of these analysis were presented as Supplementary Figure 1, in the supplementary data section. In parallel, the potential of ASC to differentiate into mesenchymal lineages including osteoblasts, chondroblasts, and adipocytes under in vitro conditions was evaluated. Osteogenic differentiation was induced by supplementing the culture medium with 10-8 M/L dexamethasone (Sigma), 5 *μ*g/mL ascorbic acid 2-phosphate (Sigma), and 10 mM/L *β*-glycerolphosphate (Sigma). To confirm the presence of calcium deposition, cultures were stained with Alizarin Red S (Nuclear, São Paulo, SP, Brazil). To induce chondrogenic differentiation, ASC were cultured in DMEM supplemented with 10 ng/mL transforming growth factor- (TGF-) *β*1 (Sigma), 50 nM ascorbic acid 2-phosphate (Sigma), and 6.25 mg/mL insulin. In order to confirm the differentiation, cells were stained with Alcian Blue pH 2.5. Adipogenesis differentiation was induced by culturing ASC in DMEM supplemented with 5 *μ*g/mL insulin, 10-6 M dexamethasone, 0.5 *μ*M isobutylmethylxanthine, and 50 *μ*M indomethacin. Cells were then stained with oil red O to confirm the presence of lipid droplets into the cell vacuoles (Supplementary Figure 2). For further characterization of ASC, immunofluorescence assays of cells cultured at the 4^th^ passage were performed. For this purpose, specific primary antibodies against CD19, CD44, CD90, CD146 (Millipore, EUA), CD73, and CD45 (Santa Cruz Biotechnology, USA), as well as fluorescent dye-conjugated secondary antibodies Alexa 488, 594, and 546 (Life Technologies), were employed. Cell nuclei were counterstained with DAPI (4′6-diamidino-2-phenylindole, Invitrogen).  Figure 1 shows these results.

### 2.4. Peritoneal Histomorphometry and Immunohistochemical Analysis

At the end of the study, after 30 days of follow-up, the animals were euthanized by IP injection of a lethal dose of sodium thiopental. Abdominal cavity was opened, and blood and tissue samples of PM were collected. Blood samples were centrifuged, and serum urea concentrations were determined using a commercially available colorimetric kit (Labtest, Brazil). Samples of the PM from the anterior abdominal wall away from the injection points were carefully dissected, immediately frozen in liquid nitrogen, and stored at -70°C for polymerase chain reaction (PCR) and multiplex analyses. Additional sections were fixed in Duboscq-Brazil solution for 45 minutes and then postfixed in buffered 4% formaldehyde solution.

PF was evaluated in sections (3 *μ*m) stained with Masson's trichrome. At least 10 digital images at 200x magnification were taken of each rat, and the thickness (*μ*m) of all photomicrographs was measured. Then, the mean peritoneal thickness from each rat was calculated [5]. For this procedure, we used digitized images and image analysis software (Image-Pro Plus Software 7.0, Media Cybernetics Inc., Bethesda, USA).

For immunohistochemical studies, PM sections were incubated with the following specific antibodies: anti-CD68 clone ED1 (Serotec, Oxford, UK), to detect macrophages; anti-CD3 (Abcam, Cambridge, MA, USA), to detect T-cells; and anti-*α*-SMA (Sigma, USA), to detect myofibroblasts. Reactions were developed using an LSAB-AP System (Dako, USA) and revealed with fast red dye (Sigma, USA). Quantitative analysis of ED1 and anti-CD3-positive cells present in the peritoneum was carried out in a blinded fashion under ×200 microscopic magnification and expressed as cells/mm^2^. The *α*-SMA staining area (%) was calculated relative to the whole peritoneal area using Image-Pro Plus 7.0 software (Media Cybernetics, Inc., Bethesda, USA).

### 2.5. Gene Expression of Fibrosis Biomarkers in PM Samples

Quantitative real-time PCR (RT-qPCR) analyses were employed to assess gene expression of some of the main fibrosis-related factors, such as collagen III, TGF-*β*, and fibronectin. For this purpose, total RNA was obtained from PM frozen samples and converted on cDNA using a commercially available kit (Promega, USA) strictly following the instructions of the manufacturer. Reactions of qPCR were conducted using the StepOnePlus Real-Time PCR System (Thermo Fisher, USA), with the following cycle program: 10 min at 95°C, followed by 40 cycles of 15 s at 95°C for denaturation, 20 s at 60°C for combined annealing, and 10 s at 72°C for extension.

### 2.6. Gene and Protein Expression of Proinflammatory Cytokines

Gene expression of cytokines such as IL-1*β*, TNF-*α*, and IL-6 were assessed by RT-qPCR, following the methods described previously. Additionally, the protein concentration of these inflammatory mediators was evaluated in the PM samples through multiplex cytokine analysis, using a commercially available kit (MILLIPLEX-EMD Millipore, Billerica, EUA), following the instructions of the manufacturer. Assays were read on the Bio-Plex Suspension Matrix System, and data were analyzed using Bio-Plex Manager version 4.0 software (Life Science, Hercules, USA). Results were expressed as pg/mg protein.

### 2.7. Statistical Analysis

Data are presented as mean ± SEM, and all statistical analyses were performed using the GraphPad Prism software, version 5.0 (GraphPad, San Diego, USA). One-way analysis of variance with pairwise comparisons according to Newman-Keuls formulation was used to compare the different experimental groups, while unpaired *t* test was used to compare different time points of each experimental group. *p* values equal to or lower than 0.05 were considered significant.

## 3. Results

### 3.1. ASC Infusion Ameliorates Body Weight Loss and Reversed Hypertension in CKD+PF Rats

All animals employed in the study showed similar body weight, systolic blood pressure, and serum urea concentration at the beginning of the protocol, before CKD or/and PF induction. As expected, the control and PF groups exhibited positive body weight gain throughout the study. Furthermore, these animals did not develop hypertension or urea retention until 30 days of follow-up. On the other hand, animals submitted to the CKD model (combined to PF or not) showed significant weight loss during the analysis period, accompanied by hypertension and uremia. As shown in Table 1 and Supplementary Figure 3, ASC treatment prevented the progression of weight loss between days 15 and 30 and reversed hypertension in the animals of the CKD+PF+ASC group.

### 3.2. ASC Treatment Prevented the Development of PF in Rats Submitted to CKD+PF

**Figure 2:**
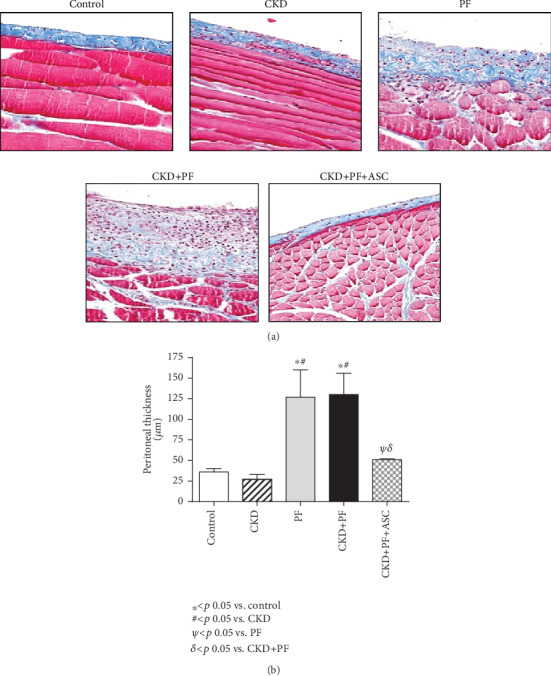
Histological features of peritoneal samples from the different groups stained with Masson's trichrome (×200) (a). The quantification of these findings was demonstrated in bar graphs (b). There were no morphological alterations in the mesothelial, submesothelial, or muscle cells in the control and CKD groups. IP CG injections induced marked submesothelial peritoneal membrane thickening, characterized by increased cellularity and collagen deposition, as can be seen in the PF and CKD+PF groups. Animals submitted to CKD+PF which received ASC infusions exhibited preserved peritoneal membrane.

The establishment of PF in the animals of the different groups was assessed by histological analyses of Masson's trichrome-stained PM samples. Illustrative microphotographs of each experimental group showed severe PM thickening and collagen accumulation in both the PF and CKD+PF groups (Figure 2(a)). The quantification of these histological findings showed that the PF and CKD+PF animals exhibited threefold greater PM than those from the control or CKD rats (*p* < 0.05), as can be seen in Figure 2(b). ASC treatment significantly prevented the development of PF in CKD+PF+ASC animals, which showed PM thickness similar to those observed in the control or CKD groups.

### 3.3. ASC Infusion Prevented PF by Reducing the Number of Peritoneal Myofibroblasts and Modulating the Expression of Genes Related to ECM Synthesis in Rats Submitted to CKD+PF

**Figure 3:**
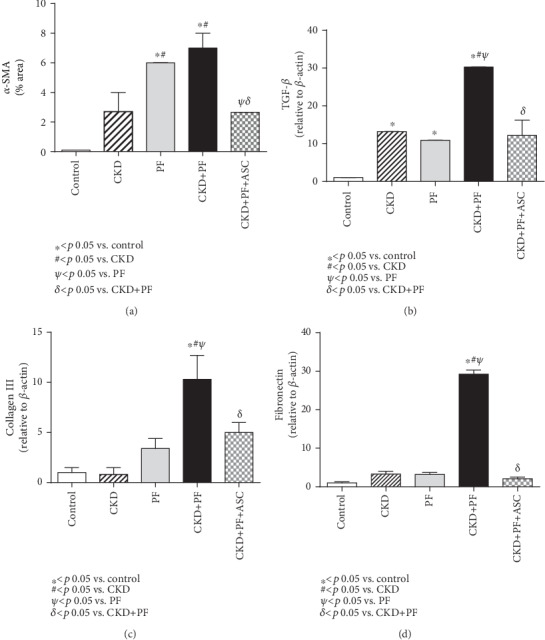
Comparative analysis of *α*-SMA expression (employed to detect myofibroblasts by immunohistochemistry) and TGF-*β*, collagen III, and fibronectin gene expressions (achieved by quantitative real-time PCR) in the peritoneal membrane of all groups. CG-induced PF was associated with a significant increase in *α*-SMA expression in both the PF and PF+CKD groups (a) that also exhibited significant overexpression of TGF-*β* (b), collagen III (c), and fibronectin (d) genes. ASC treatment markedly reduced the peritoneal percentage of *α*-SMA, as well as the expression of genes related to fibrosis.

Immunohistochemistry for *α*-SMA, a biomarker of myofibroblasts, which are ECM producer cells, strongly related to fibrogenesis, was performed in PM samples of animals of each experimental group. As shown in Figure 3(a) and Supplementary Figure 4, animals submitted to the CKD model based on adenine overload exhibited a numerical increase in the percentage of *α*-SMA in the PM, compared to the control. Corroborating our previous histological findings, the groups subjected to the PF experimental model (both PF and CKD+PF) exhibited substantial peritoneal *α*-SMA accumulation. It is noteworthy that ASC treatment significantly prevented the increase of *α*-SMA percentage in the CKD+PF+ASC animals.

Similar results were obtained from RT-qPCR analyses of PM samples to assess gene expression of some of the main profibrotic genes. PM samples of untreated animals submitted to the experimental model of combined CKD+PF showed a significant overexpression of TGF-*β*, collagen III, and fibronectin, compared with the control group. As shown in Figures 3(b)–3(d). ASC treatment significantly reduced the overexpression of TGF-*β* and collagen III and normalized the expression of fibronectin.

### 3.4. Administration of ASC Attenuated Peritoneal Inflammation

**Figure 4:**
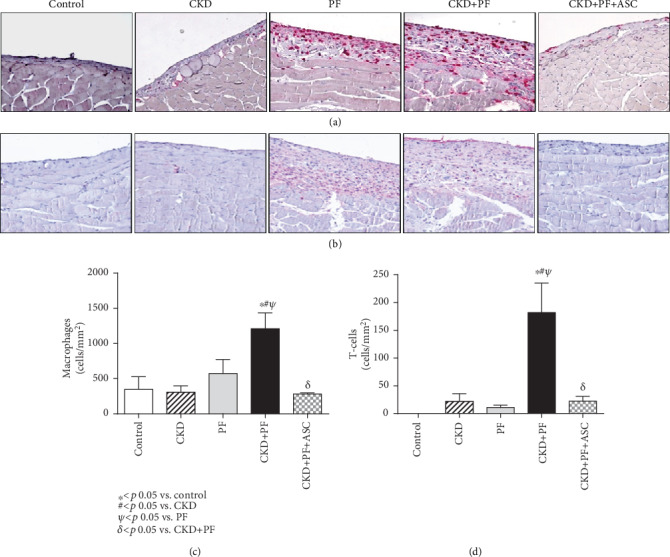
Illustrative microphotographs of peritoneal samples from the different groups submitted to immunohistochemistry for macrophage (a) and T-cell (b) detection. Associated CKD+PF induced both macrophage (c) and T-cell (d) infiltration, while ASC infusions prevented PM inflammation, completely (c, d).

In order to evaluate local leukocyte recruitment, peritoneal infiltration by macrophages (ED1+ cells) and T-cells (CD3+ cells) was evaluated by immunohistochemistry. Illustrative microphotographs are shown in Figures 4(a) and 4(b), and the quantification of these parameters is represented as bar graphs in Figures 4(c) and 4(d).

As shown in Figure 4(c) the number of macrophages detected in PM samples did not differ among animals from the control, CKD, and PF groups. Nevertheless, the combined CKD+PF model promoted a marked increase in PM infiltration by macrophages which was statistically significant when compared to the control, CKD, and PF groups (*p* < 0.05). ASC infusions completely prevented macrophage infiltration in the PM of animals from the CKD+PF+ASC group.

Peritoneal T-cells, likewise the macrophages, did not differ among the control, CKD, and PF groups, and meanwhile, were strikingly increased in the CKD+PF group. In accordance to data obtained with macrophage quantification, ASC treatment significantly reduced T-cell infiltration in the PM samples of animals from the CKD+PF+ASC group (Figure 4(d)).

**Figure 5:**
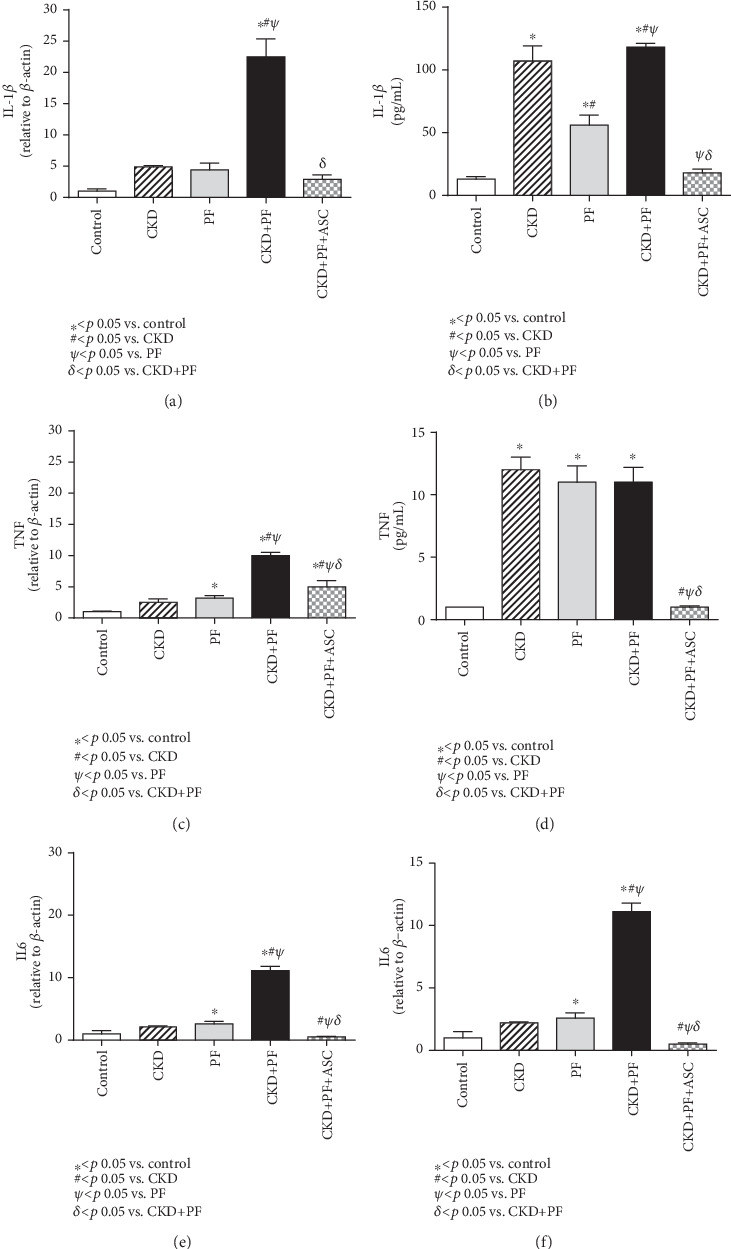
Comparative analysis of the gene (qPCR) and protein (multiplex) expressions of IL-1*β* (a, b), TNF (c, d), and IL6 (e, f) in the peritoneal membrane of the animals of each experimental group. The CKD+PF model promoted a significant increase in both gene and protein expressions of IL-1*β*, TNF-*α*, and IL6, compared to the control. ASC treatment normalized the gene and protein expressions of these three studied factors.

Additionally, we analyzed both gene and protein expressions of the following inflammatory mediators: IL-1*β*, TNF-*α*, and IL6, in the peritoneal membrane of the animals in each experimental group, as shown in Figure 5.

The CKD+PF model promoted a significant increase in both gene and protein expressions of IL-1*β*, in which the last also had an increase in the CKD and PF groups. Animals treated with ASC presented normal gene and protein expressions of IL-1*β*, compared with those observed in the control group (Figures 5(a) and 5(b)). TNF-*α* gene was overexpressed in the PM samples of animals submitted to the combined CDK+PF model, while protein levels of TNF-*α* (evaluated in PM samples by multiplex analysis) were markedly increased in the CKD, PF, and CKD+PF groups. Both gene and protein expressions of TNF-*α* were significantly reduced in the CKD+PF animals treated with ASC (Figures 5(c) and 5(d)). Accordingly, peritoneal gene and protein expressions of IL6 were notably elevated in the animals of the CKD+PF group and were completely normalized by ASC infusions (Figures 5(e) and 5(f)).

## 4. Discussion

The long-term exposure of the peritoneal membrane to bioincompatible PD solutions gradually promotes local inflammation, loss of mesothelial cells, proliferation of myofibroblasts, collagen deposition, and submesothelial thickening, leading to PF, loss of ultrafiltration capacity and, eventually, to the failure of this dialysis modality [5, 7, 13]. In this study, the intravenous administration of ASC prevented the progression of PF induced by GC in uremic rats. ASC treatment also reduced myofibroblast infiltration and attenuated the upregulated expression of profibrotic and proinflammatory genes observed in untreated animals. These findings are consistent with previous reports that studied the antifibrotic effect of stem cells in experimental PF models [14, 15]. Different from those former studies, in our experiments, we used uremic animals, and the ASC were administered by intravenous route.

As previously described, the combo experimental model of uremic CKD associated with PF employed in the present study resembles more closely the clinical and pathophysiological features observed in end-stage renal disease patients submitted to long-term PD. Besides the direct effects of CKD, such as body weight loss, systemic hypertension, and increased BUN, the animals of the CKD+PF group exhibited marked PM thickening, characterized by the submesothelial accumulation of collagen and *α*-SMA, along with peritoneal inflammation, evidenced by submesothelial macrophage and T-cell infiltration, which was statistically higher than that observed in the animals submitted only to the PF model, with no associated CKD. Additionally, local peritoneal overexpression of genes related to inflammation and fibrosis was substantially increased in the CKD+PF group compared with the PF group, thus indicating that advanced uremia aggravated the development of peritoneal inflammation in these animals [10].

The fibrous thickening and the overexpression of *α*-SMA, a myofibroblast marker, induced by GC injections in the PM were attenuated by ASC treatment. ASC administration also blocked the upregulation of profibrotic factors, notably, TGF-*β* and fibronectin. Similar to our findings, Ueno et al. demonstrated that human MSC prevented PF induced by GC in nonuremic animals. Also, the coculture of human peritoneal mesenchymal cells with human MSC resulted in a significant reduction of TGF-*β* and fibronectin mRNA expressions compared with the levels in vehicle-treated cells [14]. Since the TGF-*ß* signaling pathway plays a pivotal role in PF, it is possible that the antifibrotic effect of ASC observed in our study is mediated by TGF-*β* inhibition.

Systemic inflammation and higher levels of cytokines in the peritoneal fluid precede PF and encapsulating peritoneal sclerosis in PD patients [16]. Besides the inhibition of antifibrotic pathways, the ASC also showed a strong anti-inflammatory effect on the peritoneal membrane. Animals submitted to the combo model and treated with ASC did not present submesothelial infiltration by leukocytes, such as macrophages and T-cells. In agreement with our findings, Wang et al., using bone marrow-derived SC in a rat model of acute peritoneal adhesion, showed that intraperitoneal injections of MSC inhibited leucocyte infiltration of PM and TNF-*α* expression through paracrine mechanisms [17]. Thus, the inhibition of TNF-*α* production by ASC may account for its beneficial effect in our study.

ASC infusion also promoted significant reductions in the peritoneal gene and protein expressions of IL-1*β*, TNF-*α*, and IL-6 in the ASC-treated CKD+PF animals, compared to untreated CKD+PF rats. These findings corroborate previous reports describing MSC-induced anti-inflammatory and immunomodulatory effects. Aggarwal et al. demonstrated that purified subpopulations of human immune cells have its cytokine secretion profile altered towards a more anti-inflammatory and immunotolerant phenotype, when cocultured with MSC. According to this study, under the stimuli of MSC, dendritic cells decreased its TNF-*α* and IL-10 release, while Th1 lymphocytes reduced the IFN-*γ* expression and increased IL-4 production [18].

There are many possible mechanisms by which MSC exert its beneficial effects. The ability of MSC to migrate to the damaged tissue and differentiate into reparative cells was initially thought to occur. However, it has been recognized that paracrine factors secreted by the MSC are likely to be the main mechanism inducing tissue protection and recovery. Wang et al. showed that after IV infusion of MSC in the tail vein of rats submitted to peritoneal damage by scrapping, these cells accumulated in the lungs, liver, and spleen. No stem cells were observed in the injured peritoneum, in spite of the protective effects achieved by IV MSC infusion. These data suggest that the main biological effects of MSC infusion may be attributed more to the release of anti-inflammatory and immunomodulatory factors by these cells, than to their *in situ* differentiation [17].

Noteworthy, we found an unexpected effect of ASC treatment in blood pressure. While the uremic rats in the untreated groups (CKD and CKD+PF) showed hypertension, in the CKD+PF+ASC group, the blood pressure was similar to the control group. Future studies are needed to better understand this finding, but in an experimental model of renovascular hypertension, MSC controlled the blood pressure and suppressed the intrarenal angiotensin system [19].

## 5. Conclusions

In conclusion, we have shown that ASC treatment inhibited the progression of PF in a CG-induced PF model in uremic rats. ASC inhibited different and important mechanisms involved in peritoneal membrane modifications induced by PD, as the activation of the TGF-*β* pathway, myofibroblast proliferation, and inflammation. Our results are interesting and reinforce stem cell therapy as a perspective for the treatment of PF. However, future studies are needed before this experimental finding is translated into clinical application.

## Figures and Tables

**Table 1:** Comparative analysis of body weight (BW), systolic blood pressure (BP), and urea nitrogen (BUN) levels in the different groups at days 01, 15, and 30.

	Control	CKD	PF	CKD+PF	CKD+PF+ASC
BW (g)					
Day 01	332 ± 3	321 ± 6	319 ± 8	310 ± 4	329 ± 5
Day 15	360 ± 4^∗^	296 ± 6^∗^^†^	356 ± 8^∗^^*ϕ*^	267 ± 4^∗^^†*ϕ*¥^	279 ± 10^∗^^†¥^
Day 30	428 ± 5^∗^	239 ± 10^∗^^#†^	360 ± 8^∗^^†*ϕ*^	243 ± 11^∗^^†¥^	277 ± 8^∗^^†*ϕ*¥§^
BP (mmHg)					
Day 01	122 ± 4	129 ± 3	123 ± 2	127 ± 3	125 ± 3
Day 15	128 ± 5	164 ± 5^∗^^†^	127 ± 4^*ϕ*^	178 ± 6^∗^^†¥^	176 ± 4^∗^^†¥^
Day 30	124 ± 3	175 ± 2^∗^^†^	134 ± 6^*ϕ*^	169 ± 4^∗^^†¥^	130 ± 4^∗^^#*ϕ*§^
BUN (mg/dL)					
Day 01	51 ± 11	24 ± 13	51 ± 10	34 ± 12	60 ± 12
Day 15	55 ± 13	178 ± 16^∗^^†^	40 ± 10^∗^^*ϕ*^	185 ± 25^∗^^†¥^	169 ± 15^∗^^†¥^
Day 30	46 ± 14	307 ± 36^∗^^#†^	38 ± 12^∗^^*ϕ*^	287 ± 37^∗^^#†¥^	333 ± 24^∗^^#†¥^

Unpaired *t* test: ^∗^*p* < 0.05 vs. respective day 01, ^#^*p* < 0.05 vs. respective Day 15. ANOVA-Newman-Keuls posttest: ^†^*p* < 0.05 vs. respective control, ^*ϕ*^*p* < 0.05 vs. respective CKD, ^¥^*p* < 0.05 vs. respective PF, ^§^*p* < 0.05 vs. respective CKD+PF.

## Data Availability

All data generated or analyzed during this study are included in this published article and its Supplementary Information files.

## Supplementary Materials

Supplementary Materials Supplementary Material Figure 1: cellular characterization of mesenchymal stem cell surface markers by immunofluorescence. ASC were positive for CD44, CD90, CD146, and CD73 and negative for CD19 and CD45. Supplementary Material Figure 2: characterization of adipose-derived mesenchymal cells (ASC) employed in the study. ASC of Wistar rats in the passages P0 (A), P4(B), analysis of the capacity of ASC to differentiate into adipogenic (C), chondrogenic (D), and osteogenic lineages (E) under 10x magnification. Supplementary Material Figure 3: representative line graph of data shown in Table 1 of the main manuscript file. Comparative analysis of body weight (BW) (A), systolic blood pressure (BP) (B), and urea nitrogen (BUN) levels (C), in the different groups at days 01, 15, and 30. Supplementary Material Figure 4: illustrative microphotographs of immunohistochemistry for *α*-SMA in peritoneal samples from the different groups (×200).
