# Supplementary material for: Adipose-Derived Mesenchymal Stem Cells Modulate Fibrosis and Inflammation in the Peritoneal Fibrosis Model Developed in Uremic Rats
Source: Stem Cells Int. 2020 May 20;2020:3768718. doi: 10.1155/2020/3768718 (PMC7256710; doi:10.1155/2020/3768718)
Supplement: Supplementary Materials — Supplementary Material Figure 1: cellular characterization of mesenchymal stem cell surface markers by immunofluorescence. ASC were positive for CD44, CD90, CD146, and CD73 and negative for CD19 and CD45. Supplementary Material Figure 2: characterization of adipose-derived mesenchymal cells (ASC) employed in the study. ASC of Wistar rats in the passages P0 (A), P4(B), analysis of the capacity of ASC to differentiate into adipogenic (C), chondrogenic (D), and osteogenic lineages (E) under 10x magnification. Supplementary Material Figure 3: representative line graph of data shown in Table 1 of the main manuscript file. Comparative analysis of body weight (BW) (A), systolic blood pressure (BP) (B), and urea nitrogen (BUN) levels (C), in the different groups at days 01, 15, and 30. Supplementary Material Figure 4: illustrative microphotographs of immunohistochemistry for α-SMA in peritoneal samples from the different groups (×200). [file 3768718.f1.zip › Costalonga et al. Supplem (1).docx]

# Stem Cells International

# Adipose-derived mesenchymal stem cells modulate fibrosis and inflammation in the peritoneal fibrosis model developed in uremic rats

**Elerson C. Costalonga^1^, Camilla Fanelli^1^, Margoth R. Garnica^1^ and Irene L. Noronha^1^**

^1^ Laboratory of Cellular, Genetic, and Molecular Nephrology, Renal Division, University

of São Paulo, São Paulo, Brazil

**Correspondence:*

Irene L. Noronha, MD, PhD

Laboratorio de Nefrologia Celular, Genetica e Molecular

Faculdade de Medicina – Universidade de São Paulo

Av. Dr. Arnaldo, 455, 4o andar, Lab 4304

São Paulo, CEP 01246-903, Brasil

Tel: +5511 30618403

Fax: +5511 30618361

Email: irenenor@usp.br

## Supplementary Materials


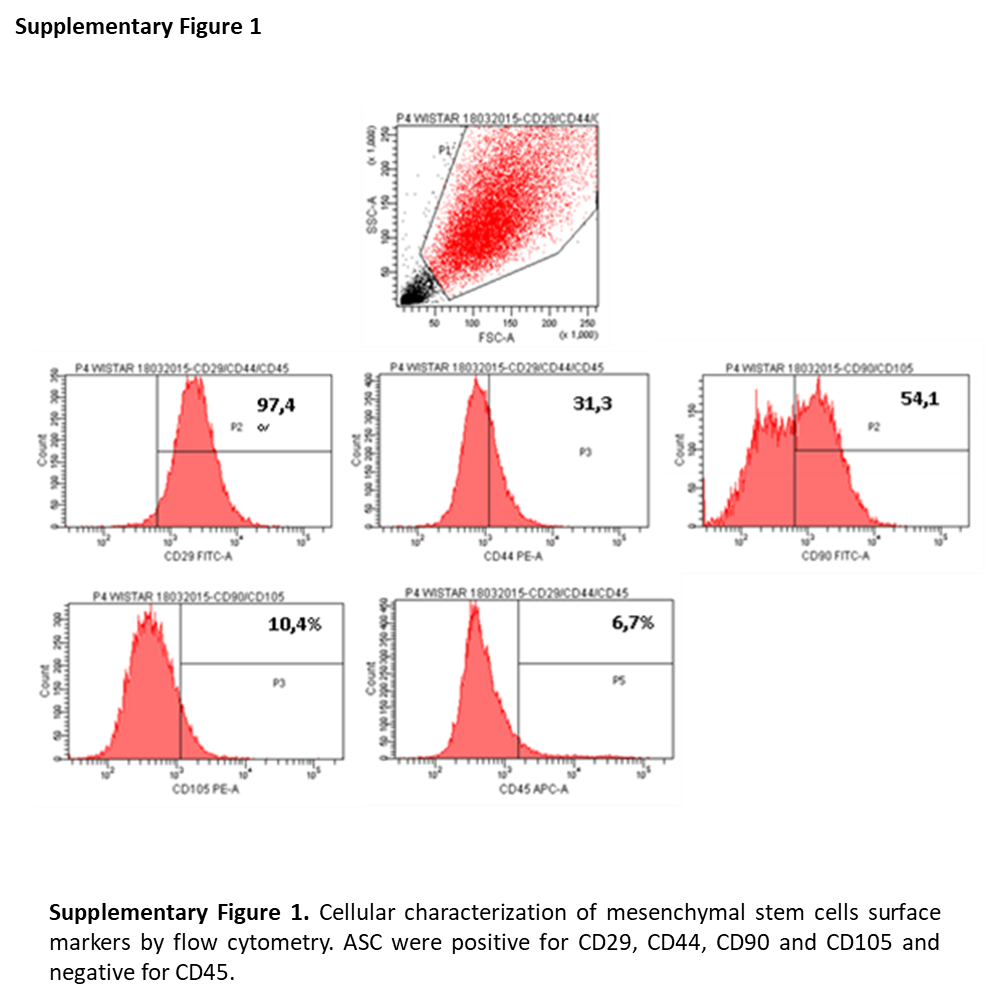


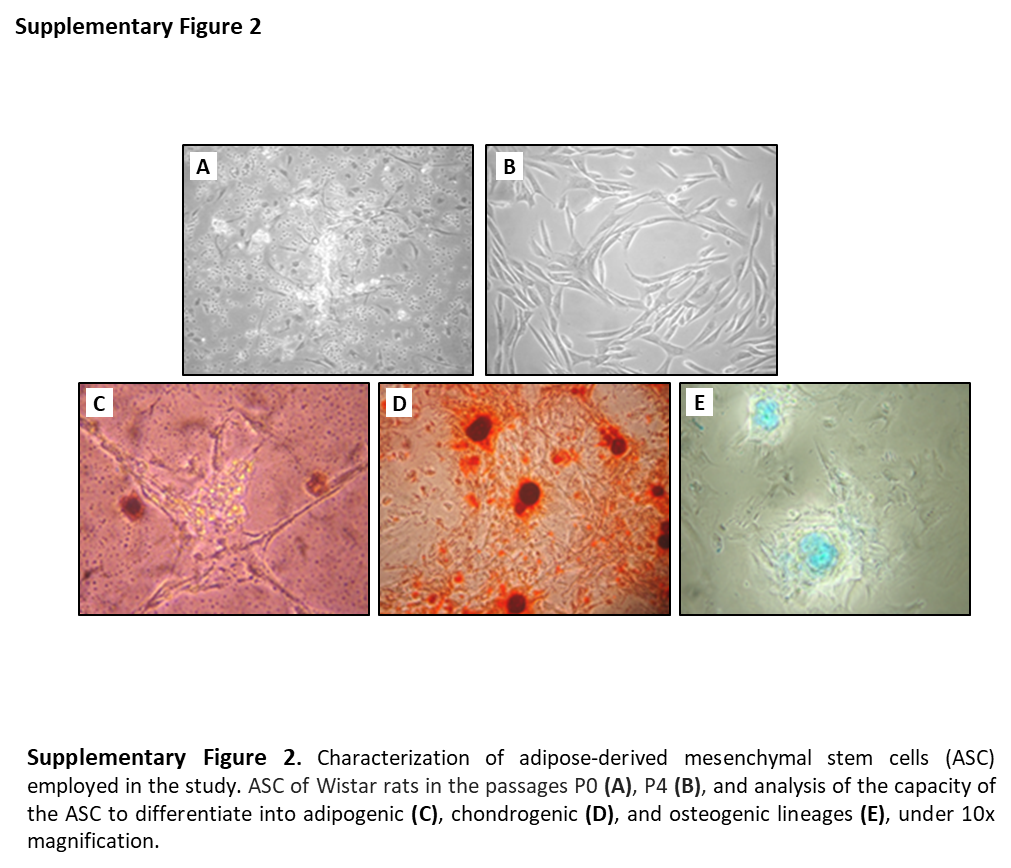


Supplementary Figure 3

**Supplementary Figure 3.** Representative line graph of data shown in the Table 1 of the main manuscript file. Comparative analysis of body weight (BW) **(A)**, systolic blood pressure (BP) **(B)** and urea nitrogen (BUN) levels **(C)**, in the different groups at days 01, 15 and 30.

**B**

**A**

**C**


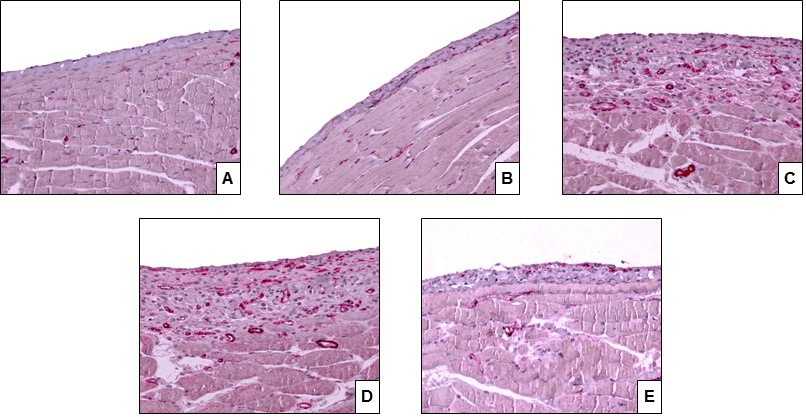

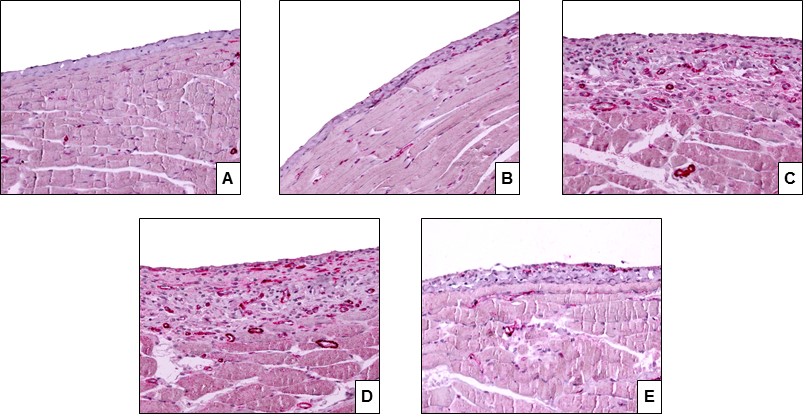

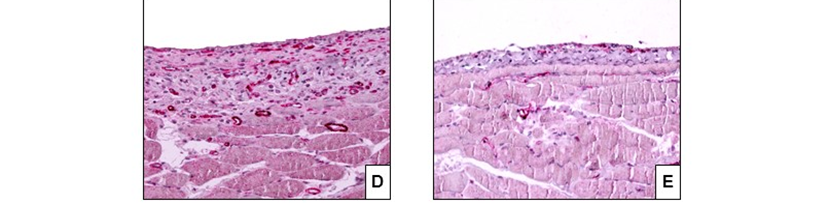


**Supplementary Figure 4.** Illustrative microphotographs of immunohistochemistry for α-SMA in peritoneal samples from the different groups (x200).


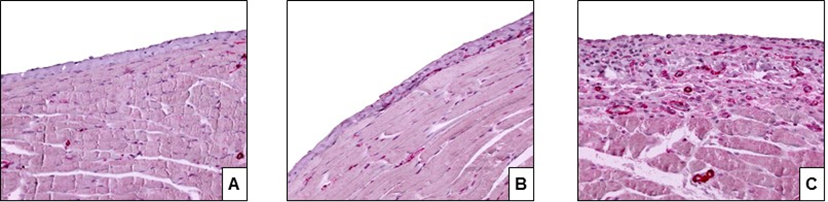

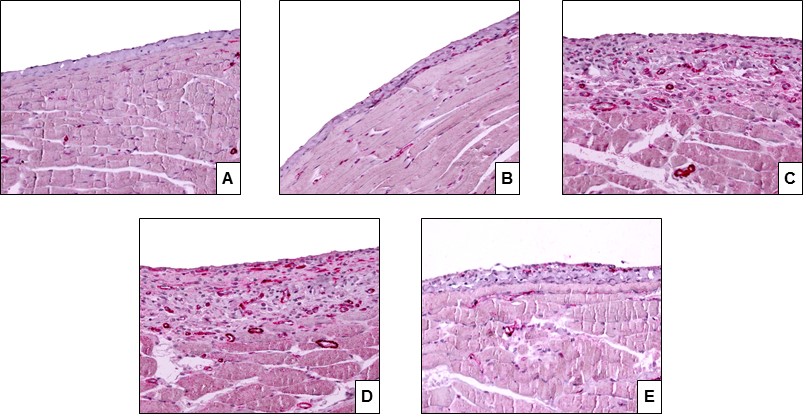


**Control CKD PF**

**CKD + PF CKD + PF + ASC**

Supplementary Figure 4
