# Supplementary figures and images for: Adipose-Derived Mesenchymal Stem Cells Modulate Fibrosis and Inflammation in the Peritoneal Fibrosis Model Developed in Uremic Rats
Source: Stem Cells Int. 2020 May 20;2020:3768718. doi: 10.1155/2020/3768718 (PMC7256710; doi:10.1155/2020/3768718)

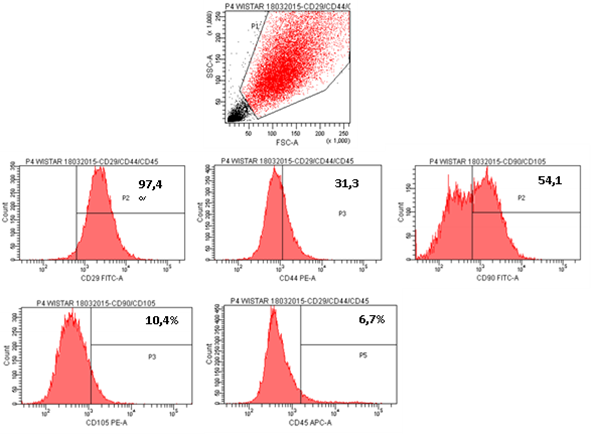

Supplement: Supplementary Materials — Supplementary Material Figure 1: cellular characterization of mesenchymal stem cell surface markers by immunofluorescence. ASC were positive for CD44, CD90, CD146, and CD73 and negative for CD19 and CD45. Supplementary Material Figure 2: characterization of adipose-derived mesenchymal cells (ASC) employed in the study. ASC of Wistar rats in the passages P0 (A), P4(B), analysis of the capacity of ASC to differentiate into adipogenic (C), chondrogenic (D), and osteogenic lineages (E) under 10x magnification. Supplementary Material Figure 3: representative line graph of data shown in Table 1 of the main manuscript file. Comparative analysis of body weight (BW) (A), systolic blood pressure (BP) (B), and urea nitrogen (BUN) levels (C), in the different groups at days 01, 15, and 30. Supplementary Material Figure 4: illustrative microphotographs of immunohistochemistry for α-SMA in peritoneal samples from the different groups (×200). [file 3768718.f1.zip › Supplementary Figure 1.docx]

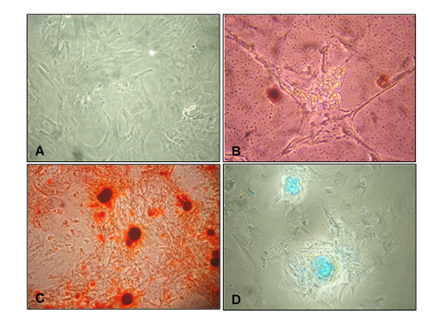

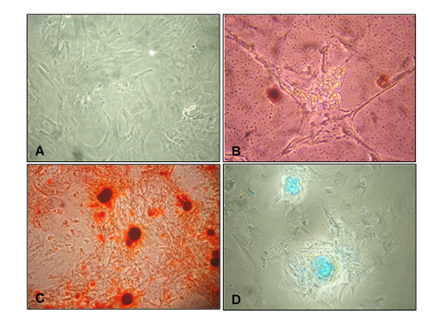


**E**


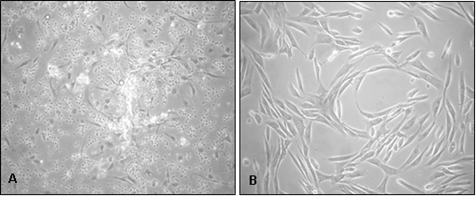


**B**


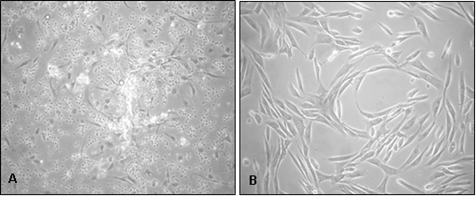


**A**


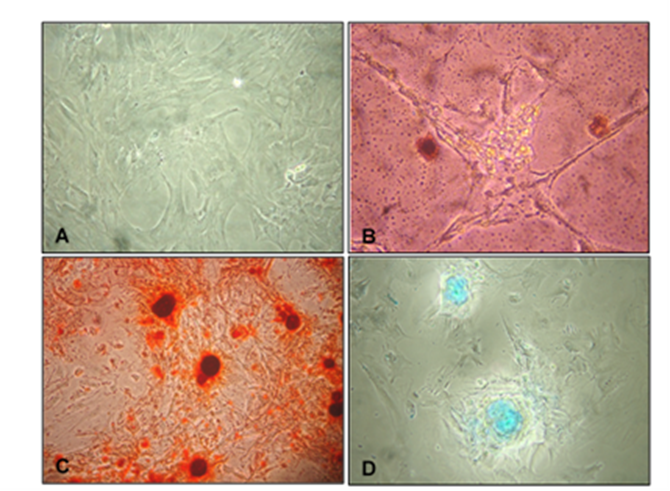


**C**

**D**

Supplement: Supplementary Materials — Supplementary Material Figure 1: cellular characterization of mesenchymal stem cell surface markers by immunofluorescence. ASC were positive for CD44, CD90, CD146, and CD73 and negative for CD19 and CD45. Supplementary Material Figure 2: characterization of adipose-derived mesenchymal cells (ASC) employed in the study. ASC of Wistar rats in the passages P0 (A), P4(B), analysis of the capacity of ASC to differentiate into adipogenic (C), chondrogenic (D), and osteogenic lineages (E) under 10x magnification. Supplementary Material Figure 3: representative line graph of data shown in Table 1 of the main manuscript file. Comparative analysis of body weight (BW) (A), systolic blood pressure (BP) (B), and urea nitrogen (BUN) levels (C), in the different groups at days 01, 15, and 30. Supplementary Material Figure 4: illustrative microphotographs of immunohistochemistry for α-SMA in peritoneal samples from the different groups (×200). [file 3768718.f1.zip › Supplementary Figure 2.docx]

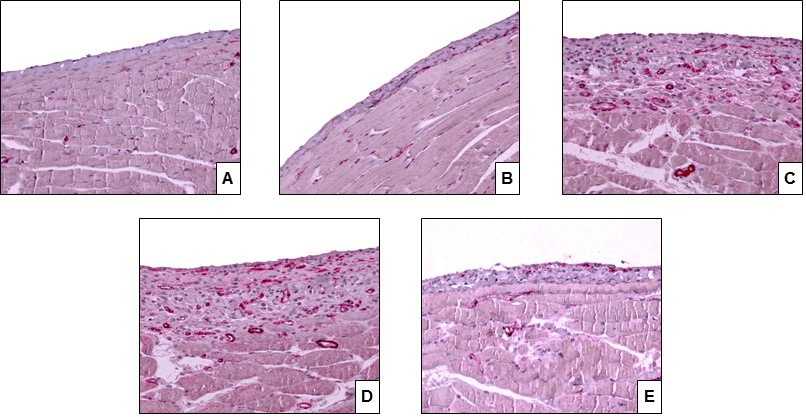

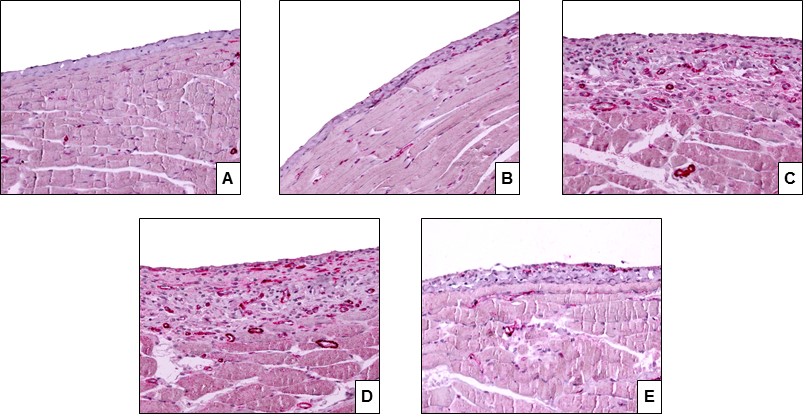

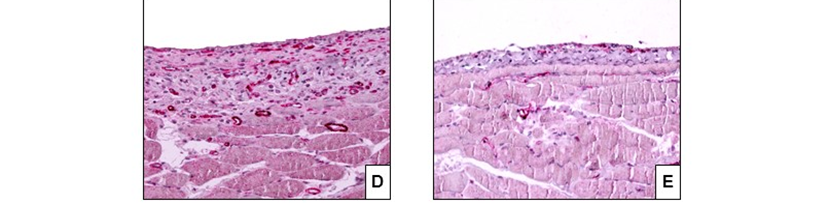

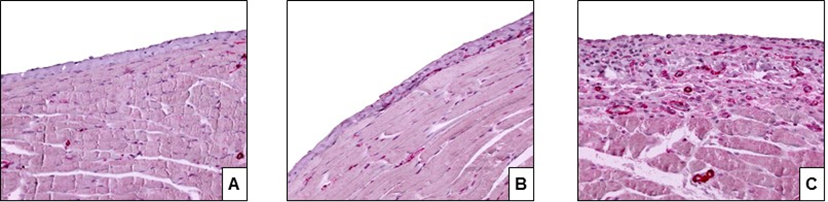

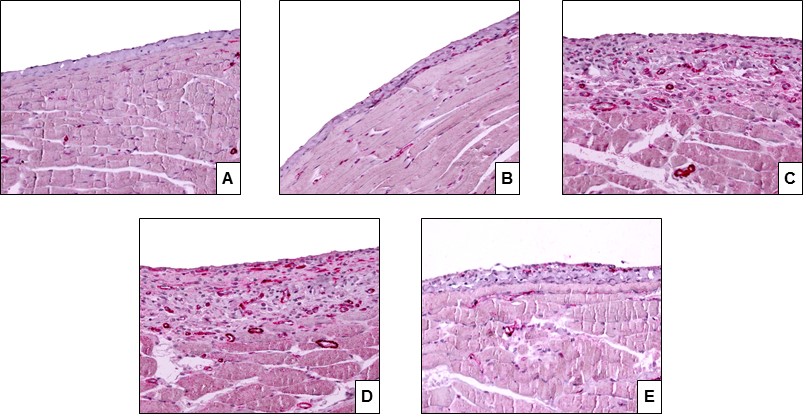


**Control CKD PF**

**CKD + PF CKD + PF + ASC**

Supplement: Supplementary Materials — Supplementary Material Figure 1: cellular characterization of mesenchymal stem cell surface markers by immunofluorescence. ASC were positive for CD44, CD90, CD146, and CD73 and negative for CD19 and CD45. Supplementary Material Figure 2: characterization of adipose-derived mesenchymal cells (ASC) employed in the study. ASC of Wistar rats in the passages P0 (A), P4(B), analysis of the capacity of ASC to differentiate into adipogenic (C), chondrogenic (D), and osteogenic lineages (E) under 10x magnification. Supplementary Material Figure 3: representative line graph of data shown in Table 1 of the main manuscript file. Comparative analysis of body weight (BW) (A), systolic blood pressure (BP) (B), and urea nitrogen (BUN) levels (C), in the different groups at days 01, 15, and 30. Supplementary Material Figure 4: illustrative microphotographs of immunohistochemistry for α-SMA in peritoneal samples from the different groups (×200). [file 3768718.f1.zip › Supplementary Figure 4.docx]
